# Virtual Reality Versus In-Person Simulation of Sepsis for Medical Students: Randomized Comparative Pilot Study

**DOI:** 10.2196/80316

**Published:** 2026-03-30

**Authors:** Lauren Medwell, Tim Old, Awais Ahmed, Victoria Holloway, Lauren McTaggart, Dafydd Morgan, Callum O'Keeffe, Jemaima Olori, Ellie Payne, Christopher Rainforth, Namritha Ramanujam, Kriti Vaidya, Chris Jacobs

**Affiliations:** 1 Undergraduate Medical Education Team, Great Western Hospitals NHS Foundation Trust, The Academy, Marlborough Road, Swindon, SN3 6BB, United Kingdom, +44 1793604020; 2 Department of Psychology, University of Bath, Bath, United Kingdom

**Keywords:** virtual reality, simulation, undergraduate, technology-enhanced learning, medical student education, high-fidelity simulation, extended reality

## Abstract

**Background:**

Virtual reality (VR) simulation—using head-mounted displays to present a computer-generated, 3D, interactive environment—may be a cost-effective alternative to in-person (IP) medical simulation training. However, studies directly comparing learning outcomes have demonstrated mixed results and mainly focused on knowledge or skill acquisition rather than integrated practice.

**Objective:**

This randomized comparative pilot study aimed to evaluate the effectiveness of VR versus IP simulation in developing sepsis management skills among final-year medical students, addressing a gap in medical education evidence.

**Methods:**

Final-year medical students at Great Western Hospital, United Kingdom, participated in both IP and VR simulation sessions featuring sepsis scenarios. Session order was randomized, determining study group assignment. Participants underwent an additional video-recorded “assessment” IP simulation of septic shock management either between or after both scheduled sessions. Questionnaires were completed between scenario completion and debriefing across all sessions. Performance was evaluated using a modified Queen’s Simulation Assessment Tool (mQSAT) by facilitating study authors across all sessions, with the assessment simulation additionally evaluated by blinded assessors. The primary outcomes included mQSAT scores, recognition of septic shock, and identification of critical care needs. Analysis of covariance was conducted to detect differences in mQSAT scores between the groups, with simulation modality as the independent variable and the number of simulations or debriefs prior to assessment as the covariate. Binary outcomes between the groups were analyzed using binomial tests.

**Results:**

A total of 32 participants were recruited and allocated to 1 of 4 groups based on completed simulation sessions prior to assessment: IP only (IP-Assess, n=10), VR only (VR-Assess, n=6), IP then VR (IP-VR-Assess, n=11), and VR then IP (VR-IP-Assess, n=5). No statistically significant differences in mQSAT scores were detected between any groups for any domain nor for the recognition of septic shock. For the recognition of need for critical care, participants who completed VR simulation only prior to assessment were more likely to recognize need for critical care than those who completed IP simulation only (3/5 vs 1/7; *P*=.01).

**Conclusions:**

This study demonstrates the feasibility of the proposed trial method and provides insight into likely effect sizes for the design of further studies. The measured learning outcomes were similar across the groups, regardless of which simulation modalities were used prior to assessment. Our study found no statistically significant differences for VR simulation versus IP simulation for the measured educational outcomes, which is reassuring for the ethical conduct of further studies comparing VR and IP simulation.

## Introduction

Virtual reality (VR) is a method of representing an immersive visual and sound sensory experience, which has the potential to mirror complex human interactions. This, coupled with its scope of user interactivity, has given researchers and educationalists significant interest in VR health care training [1,2]. VR can recreate realistic clinical scenarios [3] that promote learning in Bloom’s taxonomy of clinical knowledge [4,5], psychomotor skills [6,7], and affective attitudes [8,9]. These overlapping domains form crucial areas of learning outcomes for simulation-based education. Simulation-based education is embedded in both undergraduate and graduate curriculums across a wide range of health care professions, and the integration of technological advances has included higher fidelity manikins and immersive digital technologies [10]. Simulation combines educational theory and clinical competency within traditional and emergent technological–led practices, ultimately becoming a multifaceted pedagogy that complements teaching strategies to reduce harm to patients [11].

Despite the promise of VR training for health care professions [12], there remains a lack of robust evidence directly comparing its effectiveness to traditional simulation methods [13]. While VR offers immersive, scalable, and cost-effective training opportunities, the number of experimental studies assessing its impact on clinical skills, decision-making, and patient outcomes is limited. Many existing studies focus on user experience and engagement rather than objective performance measures, highlighting the need for more rigorous trials to establish VR’s true educational value in health care training [2]. One area of focus has been cardiopulmonary respiratory training, where VR training has been reported to be noninferior to traditional training methods of in-person (IP) simulation [14]. However, there remains heterogeneity in studies that compare VR to a control condition, whereby the content of the control differs from the intervention. Hence, this directly impacts the outcomes and interpretations.

The main objective of this study was to pilot a comparative study to evaluate the feasibility of conducting a randomized control trial comparing VR simulation of a medical emergency with traditional IP simulation methods, ensuring the alignment of educational content and incorporating an objective assessment score as the outcome measure. Second, we aimed to develop a method that would allow the comparison of these 2 differing simulation modalities, accounting for the acquisition of both knowledge and skills. Finally, we sought to quantify the effects of both IP and VR simulation on learning outcomes, inform the design of future studies, and ensure VR is not obviously inferior to IP simulation training.

## Methods

### Trial Design

As a pilot study, the full protocol was not entirely prespecified in advance. The outline methodology received local ethical approval prior to commencement of other study activities; external prospective trial registration was not pursued as this pilot study involved an intervention for health care professionals with solely education outcomes, which does not meet the International Committee of Medical Journal Editors definition of a clinical trial requiring preregistration [15]. The study was originally designed as a 1:1 randomized parallel groups trial, with an assessment simulation testing performance pre- and post-testing either IP or VR simulation teaching. A pragmatic adaptation was subsequently undertaken as follows:

We adopted a 4-parallel-arm design, with students cluster randomized to undergo an assessed simulation scenario at 1 of 4 time points relative to taught simulation sessions:

1. Assessment after IP simulation teaching only (IP-Assess group)
2. Assessment after VR simulation teaching only (VR-Assess group)
3. IP simulation teaching, then VR simulation teaching, then assessment (IP-VR-Assess group)
4. VR simulation teaching, then IP simulation teaching, then assessment (VR-IP-Assess group)

We intended to capture the added benefit of an additional session of each modality by comparing performance across arms. For example, the additional learning effect of an IP simulation could be quantified by comparing assessment simulation scores between the VR-Assess group and scores from the VR-IP-Assess group. Similarly, the additional learning effect of a VR simulation could be quantified by comparing assessment scores between the IP-Assess and IP-VR-Assess groups. Given the efficacy of VR simulation teaching is less well established than IP simulation, we targeted a ratio of 2:1 participants favoring arms informing the estimation of VR simulation efficacy (ie, the IP-Assess and IP-VR-Assess groups).

This cluster randomization process introduced incongruence between units of randomization (simulation groups) and analysis (individual participants), thus complicating the interpretation of subsequent results. However, cluster randomization is necessary when the intervention is delivered at the level of the cluster, and such incongruence is considered acceptably pragmatic research practice, particularly to facilitate research embedded within usual practice as we have done [16]. It was deemed both logistically infeasible and poorly reflective of real-world practice to deliver simulation teaching on an individual level; the statistical handling of this compromise is described in detail in the *Analytical Methods* section. This study was reported according to the Consolidated Standards of Reporting Trials (CONSORT) 2025 guidelines (Checklist 1).

### Participants

The inclusion criteria were final-year medical students on placement at Great Western Hospital (GWH). These students were time-tabled both 1 VR and 1 IP simulation session in their placement, regardless of study enrollment. Three separate cohorts, each of up to 16 students, attended during the study period for 4 weeks at a time between September and December 2023.

All students were invited to participate in this study at their placement induction and again at the beginning of their first simulation session. Students were assured that their study participation status would not affect their course progress or academic record. Participant information sheets were provided, any questions were answered, and formal written consent was taken; information sheets and consent forms are presented in our OSF repository (see [17]).

### Interventions

VR simulation sessions followed a format piloted and refined on 2 previous cohorts of final-year medical students. Three VR simulation scenarios developed by Goggleminds were available (Sepsis, v.SE1.100009; Anaphylaxis, v.AN1.100006; and Asthma, v.AS1.100006), accessed using Meta Quest 2 head-mounted displays. The sepsis simulation used was codeveloped between Goggleminds and several National Health Service training providers and has been endorsed by the Sepsis Trust [18]; it has been studied previously to review instructional design and investigate user experience, where it was demonstrated to elicit high intrinsic motivation, perceived learning, and immersion [19]. Sessions lasted 2 hours, including session prebrief and orientation to the virtual environment, 2 to 3 scenarios completed by individual students alone with other students watching via screencast to a video display, and whole group debrief following each scenario. Where 4 students attended, the final scenario was completed by 2 students concurrently, though in separate virtual environments, and with only 1 screencast. No significant software updates occurred during the study period.

IP simulation sessions were administered as per established local practice. Four to six students underwent high-fidelity simulation training in a dedicated suite, using a mix of simulated patients (played by clinical teaching fellows) and high-fidelity manikins (Ares Emergency Care Manikin, CAE). Scenarios were taken from a preapproved bank developed in our institute, all written with oversight from consultant-grade doctors from a mix of acute specialties. Sessions lasted 3 to 4 hours, including session prebrief and orientation to the simulation suite, 2 to 3 scenarios completed by students in pairs with other students watching via video link, and whole group debrief following each scenario.

Both VR and IP simulation sessions included sepsis scenarios, with adjustments made to align both as far as possible in terms of patient factors, room setup, and equipment available. Both sessions were debriefed by the same 2 faculty (LM and TO) for all participants, and debriefs included discussion of septic shock recognition and management.

### Outcomes

Learning effects of each modality were quantified using an assessment simulation, which was performed in addition to students’ usual time-tabled simulation sessions. The assessment simulation presented a simulated patient in septic shock secondary to cellulitis; full scenario details can be found in Multimedia Appendix 1.

To the authors’ knowledge, there exists no simulation performance assessment tool validated across both IP and VR modalities. To quantify differences in simulation performance across modalities, we identified the modified Queen’s Simulation Assessment Tool (mQSAT) [20]. The mQSAT uses 5 Likert-style scales ranging from inferior (1) to superior (5) performance, presented together with guide statements; 4 scales rate specific domains of primary assessment, diagnostic actions, therapeutic actions, and communication, with the final scale being a global rating. The mQSAT is validated for use in the assessment of IP simulation performance of medical students with good interrater reliability [21] and was thought prima facie to be applicable to the assessment of VR simulation.

To quantify and help account for baseline differences in participant ability, mQSAT assessments of performance in time-tabled VR and IP simulation teaching were also recorded. Two authors (LM and TO) marked all simulations, teaching and assessment, in an unblinded fashion. The assessment simulations were additionally video-recorded and double-marked by 2 blinded assessors, randomly allocated from the other study authors.

To quantify specific knowledge–based learning around the recognition and management of septic shock separate to assessment of skills or simulation performance, a questionnaire was presented to participants after the assessment simulation. The questionnaire comprised a free-text question concerning overall diagnosis; a checklist of standard actions during an A-E assessment, where participants were asked to select the actions most relevant to the scenario; and a multiple-choice question regarding the level of care required (eg, ward based vs intensive care) with justification. The full postscenario questionnaire can be found in Multimedia Appendix 1. To familiarize participants with the format prior to the assessment, the questionnaire was also provided to each participant after each scenario in time-tabled IP and VR assessment simulations, regardless of whether they had been performing the scenario themselves or watching one of their peers. The questionnaire was agreed upon between 2 of the authors (LM and TO) and designed to be usable across all scenarios across both modalities without undue prompting, yet to elicit the explicit recognition of septic shock and need for critical care input in the assessment scenario where this was achieved.

The primary outcomes were differences in the following between intervention groups:

1. mQSAT scores
2. Recognition of septic shock
3. Recognition of need for critical care

Secondary outcomes were differences in the following between intervention groups:

1. Completion of the Sepsis 6

Qualitative outcome data, including measures of participant experience and questionnaire free-text responses, were also collected and will be analyzed and reported separately.

### Sample Size

This was a pilot trial, designed in part to generate plausible effect sizes for any differences in the efficacy of IP versus VR simulation teaching; it was therefore not possible to calculate a formal sample size a priori. The decision to stop recruiting was pragmatic due to the end of the academic year.

### Randomization

**Figure 1.**
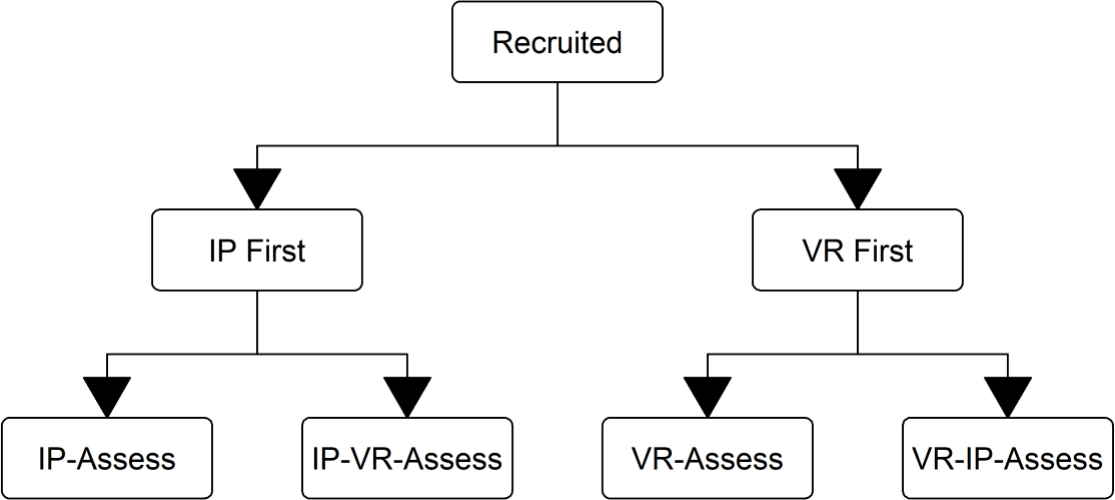
Group allocation flow diagram. IP: in-person; VR: virtual reality.

The eligible student cohort was chosen, as the existing timetable template allowed both IP and VR simulation sessions for all students in a given placement. Students attending each placement block were given a letter from A to P by their university. Students A to F were allocated to simulation group 1, students G to K to simulation group 2, and students L to P to simulation group 3. The choice of group size was linked to the capacity of the simulation suite and is reflective of typical class size for simulation teaching at the study site. The subsequent cluster randomization process and relevant R code is available in our OFS repository [17]. Briefly, room availability created natural variation between whether IP or VR simulation was time-tabled first for a given simulation group. A random number generator was used to determine the order in which available simulation slots were allocated to simulation groups, thereby randomizing simulation groups to start with either IP or VR simulation. Extra assessment simulation slots were booked either between or after both time-tabled IP and VR simulation sessions (Figure 1). All allocations were completed prior to the arrival of students at GWH, with separate authors performing allocation of random numbers (TO) and students (LM) to each simulation group.

### Analytical Methods

Analyses were finalized after the pragmatic adaptation to a 4-arm design but were fully prespecified prior to participant recruitment or data collection. Differences between mQSAT scores in the assessment simulation were statistically assessed by one-way analysis of covariance (ANCOVA), with assessment mQSAT score as the dependent variable, simulation modality being assessed as the independent variable, and number of simulations or debriefs prior to assessment as the covariate. Differences in recognition of septic shock and need for critical care were statistically assessed using binomial tests. Completion of the Sepsis 6 was compared using the Fisher exact test. Statistical testing used a significance threshold of *α*=.05; no formal correction for multiple comparisons was made. Given the nature of this study as a small-scale pilot, complete case analysis without imputation was undertaken for all analyses performed.

Pre-specified sensitivity analyses were performed to ensure study findings were robust to potential violations of assumptions. To ensure no baseline differences in students’ simulation performance, ANCOVA was repeated with baseline simulation mQSAT score (either for VR or IP training simulation session) as the covariate. Reanalysis using baseline scores as the covariate was also intended to account for issues arising from clustering effects. Versus individual-level randomization, cluster randomization inflates the variance of effect estimates in proportion to the intracluster correlation coefficient (representing how strongly individuals within clusters are related to each other), thereby decreasing statistical power [22]. Unadjusted use of methods assuming individual randomization, including ANCOVA, therefore risks an inflated type 1 error rate. ANCOVA analysis of cluster means using baseline measurements has been shown to produce similar treatment effect estimates versus the gold-standard method of mixed multilevel regression, though it is conceptually and computationally simpler [23]; given available resources, this approach was adopted as a pragmatic method of accounting for clustering effects. Finally, as all mQSAT scores for nonassessment simulations were undertaken by nonblinded study authors (LM and TO), to assess bias in scoring from nonblinded versus blinded mQSAT scoring, interrater agreement was quantified by the Krippendorff *α*.

### Ethical Considerations

The study protocol was granted ethical approval by the Swindon Academy Medical Education Research Committee (reference: LMTO0823). An outline methodology was drafted and submitted to peer review as part of obtaining local ethical approval; relevant documentation is available via our OSF repository [17]. All students were time-tabled for both VR and IP simulations regardless of study participation. Students were assured that their study participation status would not affect their course progress or academic record and that they could withdraw at any time; participation was entirely voluntary, with no financial or other compensation offered. Participant information sheets were provided, any questions were answered, and formal written consent was taken; information sheets and consent forms are presented in our OSF repository [17]. Potential risks and mitigations were considered and communicated to participants within written information and verbal prebriefs prior to simulations. Video recordings were performed using Microsoft Teams linked to a secure Trust account of one author (TO) and were only available to the research team otherwise through sharing secure links to individual videos on a named-person basis to relevant study staff as required. All digital participant data including video recordings were held on secure Trust computer systems, with physical forms stored in a secure office; all data were held for the duration of the study and destroyed on study completion.

## Results

### Participant Recruitment and Flow

**Figure 2.**
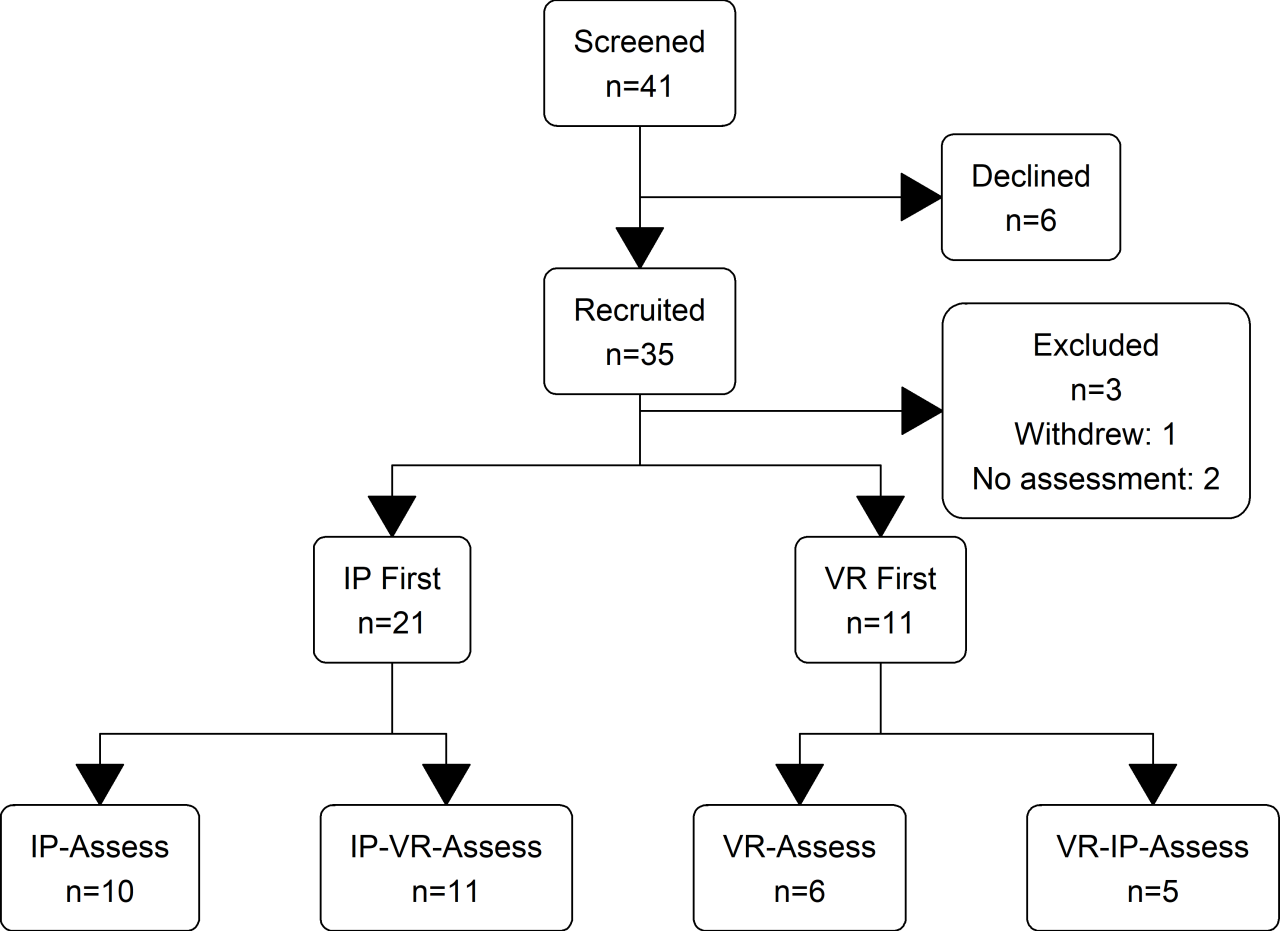
CONSORT (Consolidated Standards of Reporting Trials) flow diagram. IP: in-person; VR: virtual reality.

A total of 41 students were approached, and 35 participants were recruited between September 25 and December 22, 2023. One participant withdrew, and 2 participants did not attend the assessment simulation due to illness. There were therefore 32 participants in the final analysis for each of the primary and secondary outcomes; participant flow is summarized in Figure 2. All 32 participants otherwise completed all simulation sessions as scheduled. Recruitment was stopped due to the end of eligible final-year student placements at GWH for the academic year.

### Baseline Data

Baseline characteristics are summarized in Table 1, and the baseline experience of simulation training and VR use is summarized in Multimedia Appendix 2 in Tables S1-S3. Baseline characteristics were broadly comparable across the groups with respect to age, sex, proportion of graduate-entry students, previous simulation experience, and previous VR experience.

**Table 1.** Baseline characteristics—age and proportion of graduate-entry students.

Study group	Participants, n	Age (y), mean (SD)	Graduate-entry students, n (%)	Female, n (%)
IP-VR-Assess^a^^b^	11	25.6 (2.7)	2 (18)	5 (46)
VR-IP-Assess	5	24 (1.4)	1 (20)	1 (20)
IP-Assess	10	23.4 (0.5)	0 (0)	5 (50)
VR-Assess	6	25 (2.8)	2 (33)	4 (67)

a IP: in-person.

b VR: virtual reality.

### Outcomes and Estimation

#### Primary Analyses

**Figure 3.**
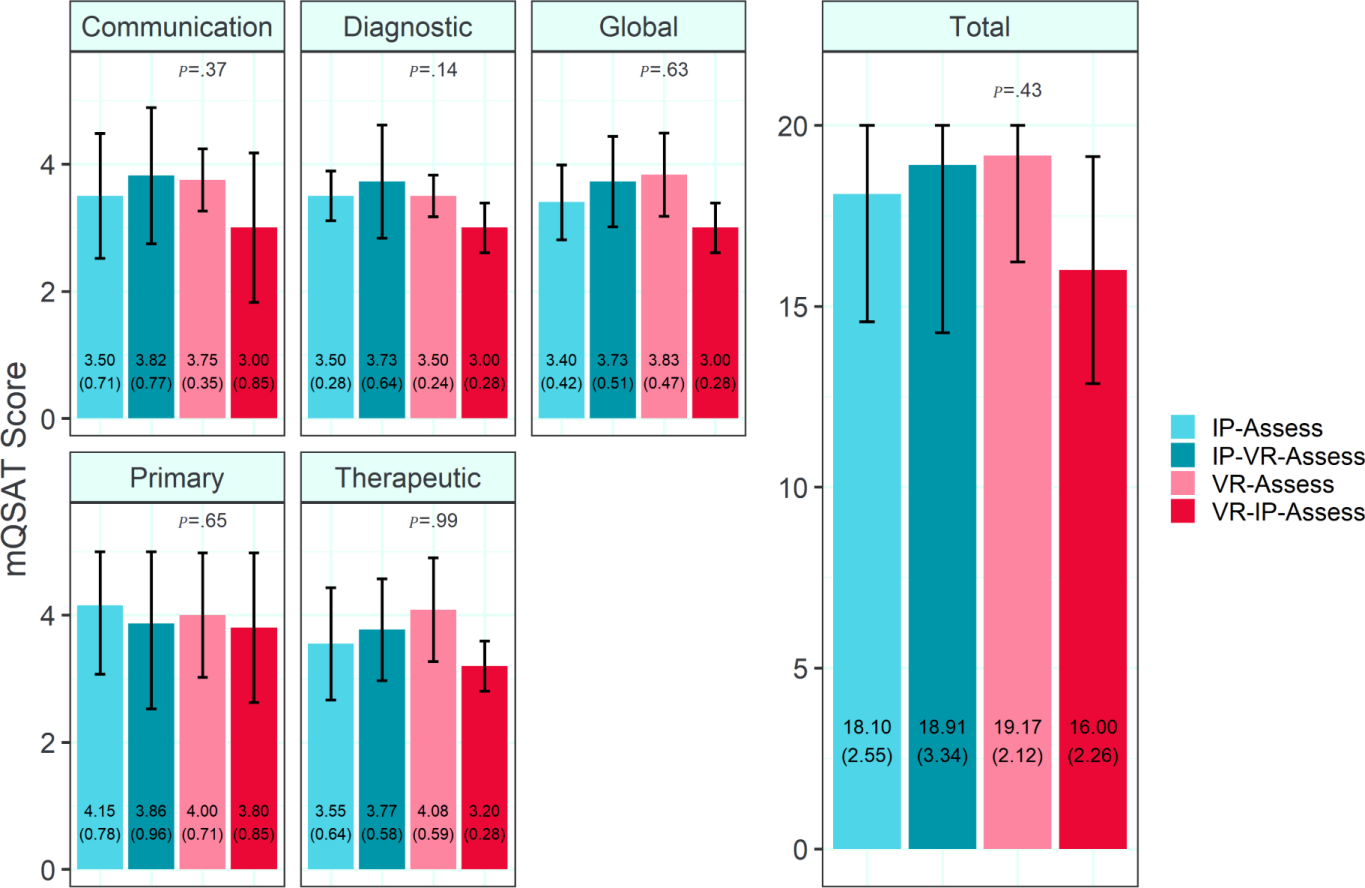
Mean(SD) Modified Queen’s Simulation Assessment Tool (mQSAT) scores by study group. Error bars are 95% confidence interval. IP: in-person; VR: virtual reality.

For mQSAT scores (n=32), no statistically significant differences were observed between any of the study groups for any domain by ANCOVA analysis. The results are summarized in Figure 3.

For the recognition of septic shock (n=32), no statistically significant differences were observed between groups by binomial test. Results are summarized in Table 2.

**Table 2.** Recognition of septic shock and need for critical care referral by study group.

Study group	Participants, n	Prior debriefs, n	Septic shock recognized^b^, n	Septic shock *P* value^a^	Critical care recognized^b^, n	Critical care *P* value^a^
IP-Assess^c^	10	1	7	.23	1	.01
VR-Assess^d^	6	1	5	—^e^	3	—
IP-VR-Assess	11	2	7	.70	2	>.99
VR-IP-Assess	5	2	3	—	0	—

a *P* value by binomial test.

b Agreed by both blinded reviewers.

c IP: in-person.

d VR: virtual reality.

e Not applicable.

For recognition of need for critical care (n=32), a statistically significant difference was noted between the groups in participants who had received only 1 debrief (ie, 1 simulation session) prior to assessment. Participants who completed only 1 VR simulation and debrief prior to assessment were more likely to recognize need for critical care than those who completed only IP simulation and debrief (3/5 vs 1/7; *P*=.01). The results are summarized in Table 2.

#### Secondary Analyses

**Figure 4.**
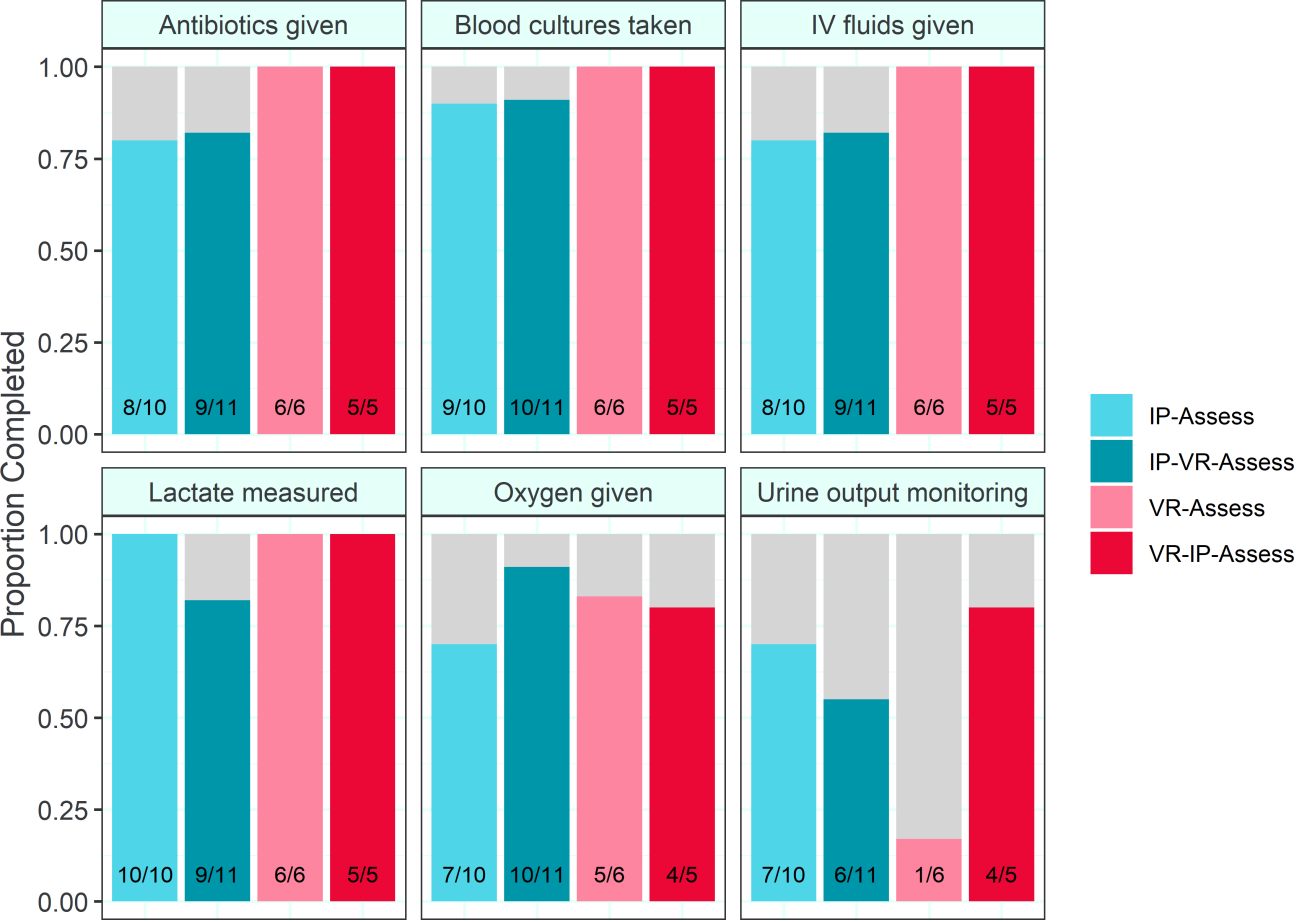
Proportion of Sepsis 6 components completed by study group. IP: in-person; VR: virtual reality.

For the completion of the Sepsis 6 (n=32), no differences in performance were observed between any groups for any component by the Fisher exact test. The results are summarized in Figure 4.

#### Ancillary Analyses

The results of prespecified sensitivity analyses are reported in full in Multimedia Appendix 3.

Using first mQSAT scores as the covariate in ANCOVA found no significant differences between the study groups (n=32; Table S1 in Multimedia Appendix 3).

Using either unblinded reviewers’ assessment mQSAT scores or all 4 reviewers’ assessment mQSAT scores did not change conclusions, finding no significant differences between the study groups (n=32; Tables S2 and S3 in Multimedia Appendix 3).

Using a more lenient criterion of any of 4 reviewers marking the recognition of septic shock or need for critical care did not change conclusions, finding no significant differences between the study groups (n=32; Table S4 in Multimedia Appendix 3).

The Krippendorff α indicated slight-to-moderate agreement between reviewers (n=4; range −0.155 to 0.491; Table S5 i-iv in Multimedia Appendix 3).

### Harms

No unintended harms, data breaches, or other unintended effects were noted during the study. Qualitative feedback was collected from participants, which will be fully reported separately.

Some noted motion or cybersickness, which is a known occurrence in VR settings. This was considered in our risk assessment and communicated during participant prebriefings. Two participants reported motion sickness during sessions, leading to a brief pause and the option to stop completely. With small adjustments, such as completing the simulation in a seated rather than ambulatory position, participants were able to continue and complete the full simulation.

## Discussion

### Principal Findings

This pilot study found no statistically significant differences in overall simulation performance between medical students who received VR simulation training versus those who received traditional IP simulation training for sepsis management. The objective assessment mQSAT scores across all domains (primary assessment, diagnostic actions, therapeutic actions, communication, and global rating) showed comparable performance regardless of the simulation modality used prior to assessment. The only statistically significant finding was that students who completed a single VR simulation session prior to assessment were more likely to recognize the need for critical care compared to those who completed a single IP simulation session (3/5 vs 1/7; *P*=.01). These findings showed no clear differences between VR simulation and traditional IP simulation for teaching sepsis recognition and management to final-year medical students. While this is encouraging, this study was neither designed nor powered to conclude noninferiority with certainty. This exploratory pilot study does suggest feasibility of an experimental design and may be beneficial to the design of a larger piece of research to consider noninferiority.

### Comparison With Prior Work

Our findings align with the growing evidence base suggesting VR may be a viable alternative to traditional simulation methods in health care teaching. A systematic review and meta-analysis of VR applications in health care education found that VR use could achieve similar or better knowledge acquisition and skills scores as well as producing greater satisfaction and confidence compared to conventional teaching methods across multiple specialties [12]. The review identified wide heterogeneity and need for more studies using objective evaluation tools. Our study piloted directly comparing performance outcomes between modalities using a validated assessment tool, although formal validation of this tool for use in VR would be advisable prior to use in a larger study.

Studies have shown mixed results when comparing VR to traditional simulation with insufficient evidence to endorse 1 modality. A systematic review reported on 15 randomized control trials, with 2 demonstrating traditional simulation superiority, 4 showing VR simulation was superior, and 8 with comparable outcomes [13]. However, most of these studies focused on learning reactions or knowledge tests, arguably not the main intended learning outcome of simulation. Focusing on the “shows how” level of the pyramid of Miller [24], several studies demonstrated comparable outcomes between simulation modalities. A randomized control trial on managing status epilepticus found equivalent times to critical actions for VR and traditional simulation training [25]. The noninferiority of VR simulation was also found for nursing students learning ABCDE (Airway Breathing Circulation Disability Exposure) approach examinations [26] and for learning appropriate triage of casualties within mass casualty incidents [27]. Additionally, a multisimulation study comparing a package of 4 VR versus 4 traditional simulations for nursing students found that the VR group had significantly better knowledge outcomes and equivalent practical test (Objective Structured Clinical Examination) scores [28]. Our findings add to the growing body of evidence that VR simulation may provide equivalent outcomes to traditional simulation when teaching these integrated performance skills.

Much research comparing VR with traditional teaching methods using objective findings has primarily been on surgical skills acquisition [29] and resuscitation [14]. Systematic reviews on VR simulation to teach resuscitation found promising overall benefit for health care professions [14] and laypeople [30]. For non–health care professionals, a study demonstrated significantly better cardiopulmonary resuscitation training across several metrics within the VR group with learning retained 12 months post-training [31]. A systematic review of VR in surgical training found that VR showed benefits in multiple areas, including procedural times and postintervention scoring [32]. Our findings on sepsis management extend these results to another critical clinical scenario, suggesting that VR may effectively teach the recognition and management of septic shock and also the procedural steps required for the correct management and escalation of care.

Regarding specific outcomes for sepsis training, it has been shown for VR simulation-based education that sepsis management can be learned, with improvements in knowledge and interpersonal skills development [33]. Similarly, earlier work on VR sepsis simulation development evidence high perceived learning levels and optimum cognitive load [19]. Our study builds on this by demonstrating that VR might achieve similar recognition of sepsis and critical care needs. This is particularly relevant to VR’s potential cost-effectiveness and scalability compared to traditional simulation methods [34-36].

The slightly higher recognition of critical care needs in the VR-only group compared to the IP-only group is an interesting finding that warrants further investigation. It is possible that this is a false-positive signal due to multiple comparisons made, though plausible explanations for a potentially causal relationship are also apparent. VR’s immersive environment may enhance situational awareness and clinical decision-making, particularly for time-critical scenarios [37]. Indeed, exposure to a VR simulation in pediatrics significantly increased the recognition of respiratory distress [38] with a subsequent study demonstrating most participants found that the modality accurately depicted a decompensating patient [39]. Alternatively, the standardized nature of VR scenarios might provide more consistent exposure to specific clinical cues compared to IP simulations, which can vary based on facilitator and simulated patient performance [40]. A randomized control study highlighted repeatability as one of the positives of VR, which may have contributed to VR’s positive study outcomes [28]. In the first instance, however, this finding should be interpreted as hypothesis-generating only, and further study would be needed to fully investigate the differential recognition of critical care need between these simulation modalities.

Our finding of only slight-to-moderate interrater reliability (Krippendorff *α*=−.155 to 0.491) for mQSAT scores is consistent with existing literature on simulation assessment challenges [41], although some studies report higher levels of interrater reliability [42]. A validity assessment of score inferences benefits from scoring and item-total correlations. While the mQSAT has demonstrated good reliability in previous studies with IP simulation [21], our findings suggest that assessment tools may need further validation across different simulation modalities.

Regarding procedural skills, we found no significant differences in Sepsis 6 bundle completion rates between the groups. Research has demonstrated that procedural skills can be effectively taught through various simulation modalities when coupled with structured debriefing [43,44]. This highlights the importance of the educational approach surrounding the technology rather than the technology itself, a finding echoed within medical education literature [45,46]. Research regarding virtual simulation specifically notes the importance of debrief regardless of the modality used, as it is central to learning outcomes [34,47]. Our findings reinforce this concept, suggesting that the structured debriefing process used in both IP and VR modalities influences the outcomes. This aligns with pedagogical frameworks emphasizing that technology serves merely as a vehicle for instruction, while learning outcomes are primarily determined by instructional design, facilitation quality, and debriefing approaches. The comparable performance across modalities in our study, despite the substantial technological differences between VR and IP simulation, underscores this principle.

### Limitations

Our study had several limitations. The small sample size (n=32) across the 4 groups limited statistical power for detecting subtle differences, with imbalanced group sizes further constraining our analysis. The use of unequal group sizes would not be expected to bias effect estimates but reduces the precision of estimates for the smaller groups [48]. In our study, smaller sizes of arms estimating the learning effects of IP simulation may reduce our ability to distinguish learning differences between our cohort and those previously reported in the literature, impacting external validity. While pragmatically necessary, the cluster randomization method of a small sample potentially introduced selection bias despite broadly comparable baseline characteristics between the groups. The pilot nature of this comparative study, while useful for developing future research, does mean only limited conclusions can be drawn.

Our study did not use a classical pretest-posttest design, which may impact the ability to ascribe observed outcomes directly to the interventions studied. It has previously been suggested that a pretest may be undesirable due to, for example, influencing learning during the intervention, or familiarity with the assessment; however, it may be warranted when pretesting is an integral part of the intervention, when using a nonrandomized design, or for small sample sizes of less than 40 per interventional arm, in order to account for baseline differences between study groups [49]. We anticipate that our cluster randomization should have at least reduced, even if not entirely eliminated, baseline differences between the groups. Our sensitivity analysis using First mQSAT scores as the covariate in ANCOVA was designed to account for any residual baseline differences in simulation performance despite cluster randomization; this analysis did not change conclusions.

The mQSAT tool, though validated for IP simulation assessment, has not been previously validated for VR simulation scenarios. Modest interrater reliability suggests that assessment tools designed specifically for cross-modality evaluation may be needed. The validation of this tool within VR should be undertaken before use in larger studies to ensure it is appropriate, especially regarding areas that might be approached differently in VR versus IP simulation, such as communication. Additionally, our assessment focused on immediate performance without longitudinal follow-up, preventing conclusions about knowledge retention or transfer to clinical practice. The potential novelty effect of VR technology may have influenced student engagement and performance, which may not be found on follow-up assessment. However, there is initial evidence that some retention is found on longitudinal follow-up of VR-taught clinical skills 2 weeks [50] and 1 year later [51].

As a single-center study with a homogeneous population of final-year medical students, our findings have limited generalizability to different learner populations, institutions, or health care systems with varying simulation resources and curricula.

### Implications for Practice

Despite these limitations, our findings have several important implications for medical education. The comparable performance between VR and IP simulation groups adds to the evidence base that VR may be a viable alternative for clinical management when traditional simulation resources are limited or inaccessible. This could be particularly valuable for institutions with geographic, financial, or staffing constraints that limit access to high-fidelity simulation centers.

The higher recognition of critical care needs in the VR-only group is an interesting finding requiring further investigation but could represent the more life-like appearance of a sick patient constructed in VR compared to the normally well-looking IP simulation representative. Educational designers might consider using VR specifically for scenarios that emphasize the recognition of deterioration and escalation of care. The similar completion rates of Sepsis 6 components across groups suggest that procedural aspects of care can be taught effectively with either modality. However, institutions should consider the specific educational objectives, available resources, and learner characteristics when selecting between simulation approaches.

### Future Research

This pilot study provides valuable data for designing future larger-scale trials. A fully powered noninferiority trial with a larger sample size would allow more definitive conclusions about the relative effectiveness of VR versus IP simulation for clinical education. Such a trial should include longitudinal follow-up to assess knowledge retention and, ideally, impact on clinical practice.

Future research should also explore the optimal integration of VR and IP simulation within a comprehensive curriculum. Rather than viewing these as competing modalities, a blended approach might leverage the strengths of each method for different learning objectives or at different stages of training. VR may be particularly valuable for independent practice and reinforcement after initial IP simulation training. Finally, cost-effectiveness analyses comparing VR and IP simulation would further inform educational policy and resource allocation decisions. While VR has potential scalability advantages, the initial development and technology costs should be weighed against the long-term benefits.

### Conclusions

This pilot study found no significant differences in sepsis management performance between medical students trained using VR versus IP simulation, with the exception of slightly better recognition of critical care needs in the VR-only group. These findings indicate that VR simulation performed similarly to IP simulation for teaching sepsis management to final-year medical students, though larger studies are needed for definitive conclusions. As simulation technology continues to evolve, educational approaches that strategically integrate various modalities based on specific learning objectives, available resources, and learner needs are likely to be most effective. VR simulation shows promise as a complementary or alternative approach to traditional simulation methods, potentially expanding access to high-quality simulation education across diverse educational settings.

## Supplementary material

10.2196/80316 Multimedia Appendix 1 Scenarios and questionnaires.

10.2196/80316 Multimedia Appendix 2 Baseline testing.

10.2196/80316 Multimedia Appendix 3 Sensitivity analyses.

10.2196/80316 Checklist 1 CONSORT checklist.

## Abbreviations

ABCDE: Airway Breathing Circulation Disability Exposure
ANCOVA: analysis of covariance
CONSORT: Consolidated Standards of Reporting Trials
GWH: Great Western Hospital
IP: in-person
mQSAT: Queen's Simulation Assessment Tool
VR: virtual reality
