# Supplementary material for: Virtual Reality Versus In-Person Simulation of Sepsis for Medical Students: Randomized Comparative Pilot Study
Source: JMIR Med Educ. 2026 Mar 30;12:e80316. doi: 10.2196/80316 (PMC13035032; doi:10.2196/80316)
Supplement: Multimedia Appendix 1 [file mededu-v12-e80316-s001.docx]

Multimedia Appendix 1 - Scenarios and Questionnaires

**C.1 Assessment Simulation – Simulated Patient Brief:**

Sam Brookes

29y, 22/6/1994

You fell off you bike last week coming down a hill, landed on left leg. Not too bad at the time. Unsure if any broken skin as was wearing thick jeans, maybe minor graze if anything.

Past couple of days, have noticed left leg increasingly sore, also getting redder.

Woke up this morning feeling generally achey, tired and sweaty, (clammy rather than feverish if asked directly). Saw GP briefly today as same day appointment who sent in

No PMHx/reg meds/allergies

Mother had T2DM, otherwise no FHx

Civil engineer, lives alone. Keen cyclist.

EtOH: 1 bottle wine/week

Smoking: social only as student

Systems r/v: hasn't passed urine today (don’t volunteer unless asked), otherwise nil

Exam:

Gen: sweaty and unwell. Sat in chair. Cannula in situ

A: NAD

B: RR slightly up (acidotic). Chest clear, sats OK

C: hypotensive/tachycardic, not fluid responsive. CRT 2-3s.

D: appears drowsy/tired, otherwise NAD

E: Left leg red anterolaterally, very painful to touch. No urine if catheterised

Bloods:

Raised inflammatory markers, compensated lactic acidosis on gas

ECG: sinus tachy

No urine for dip.

Progression:

Remains tachycardic/hypotensive despite fluids/abx

If not noted, nurse to prompt

End scenario after discussion with SpR (any of med/surg/ITU). Med SpR calls at 14 mins if not yet escalated


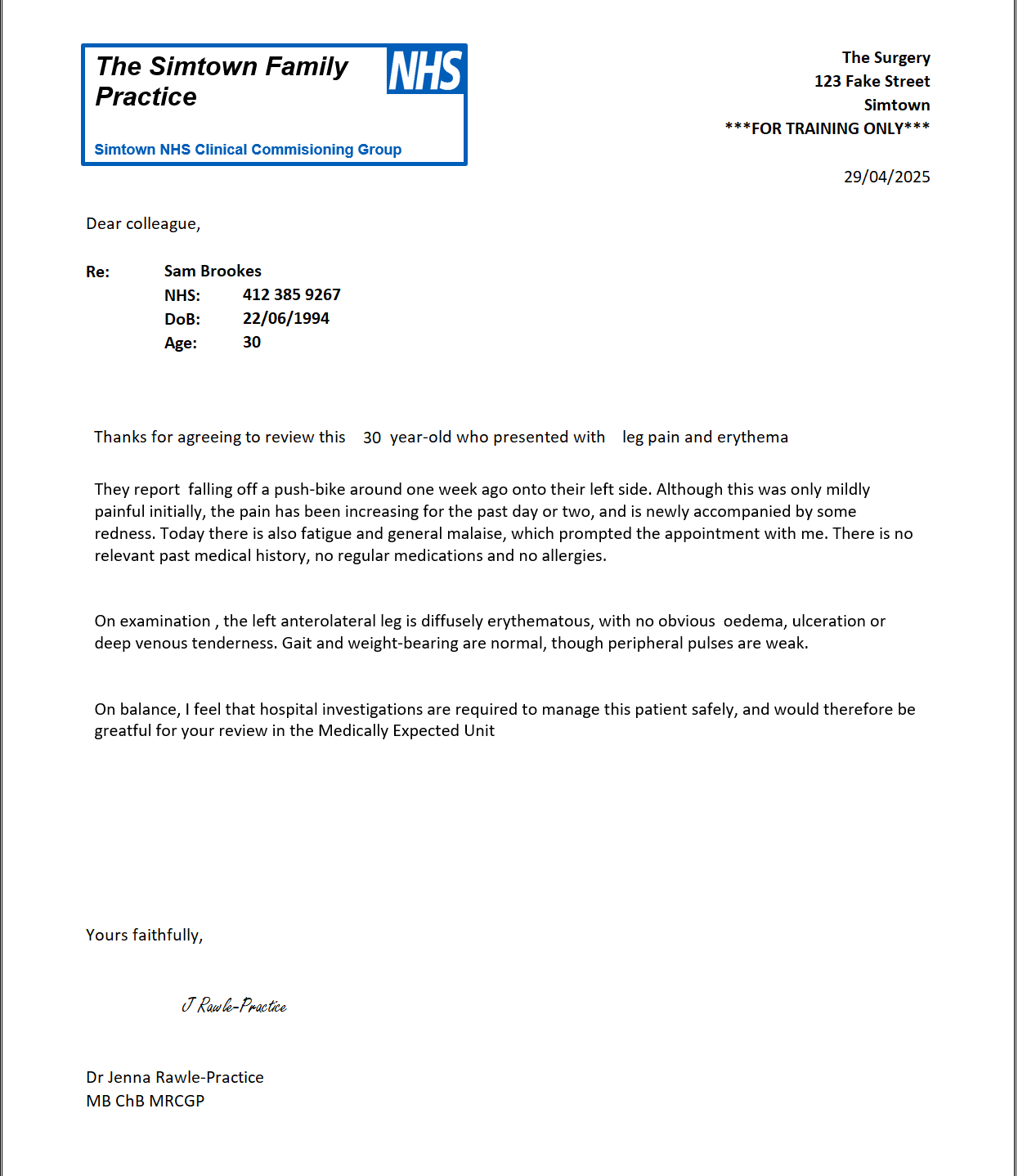
**C.2 Assessment Simulation – Simulated Patient Referral Letter:**

**C.3 Assessment Simulation – Simulated Patient Triage Note:**

**
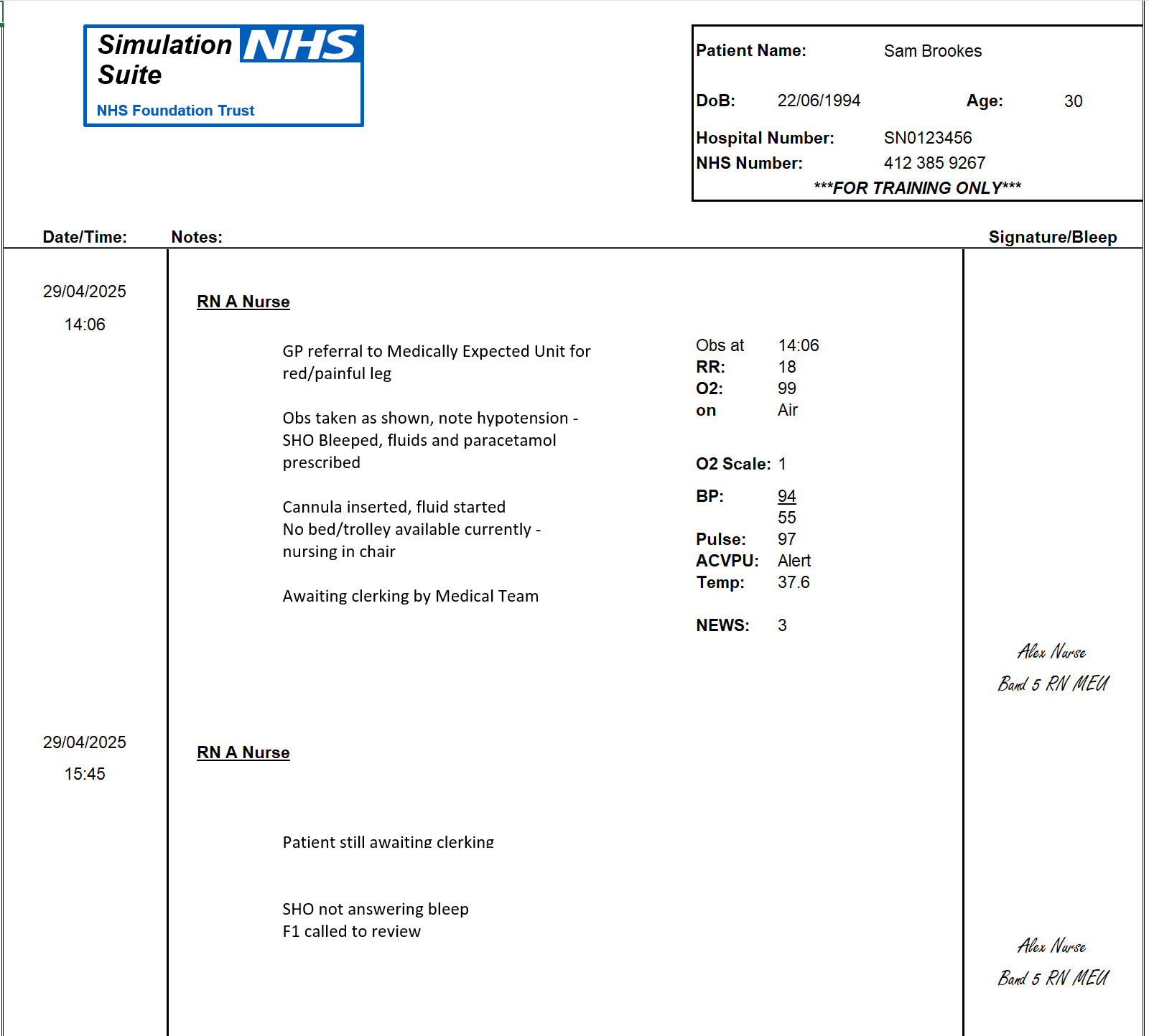
**

**C.4 Assessment Simulation – Simulated Patient Observations Pre-Fluids:
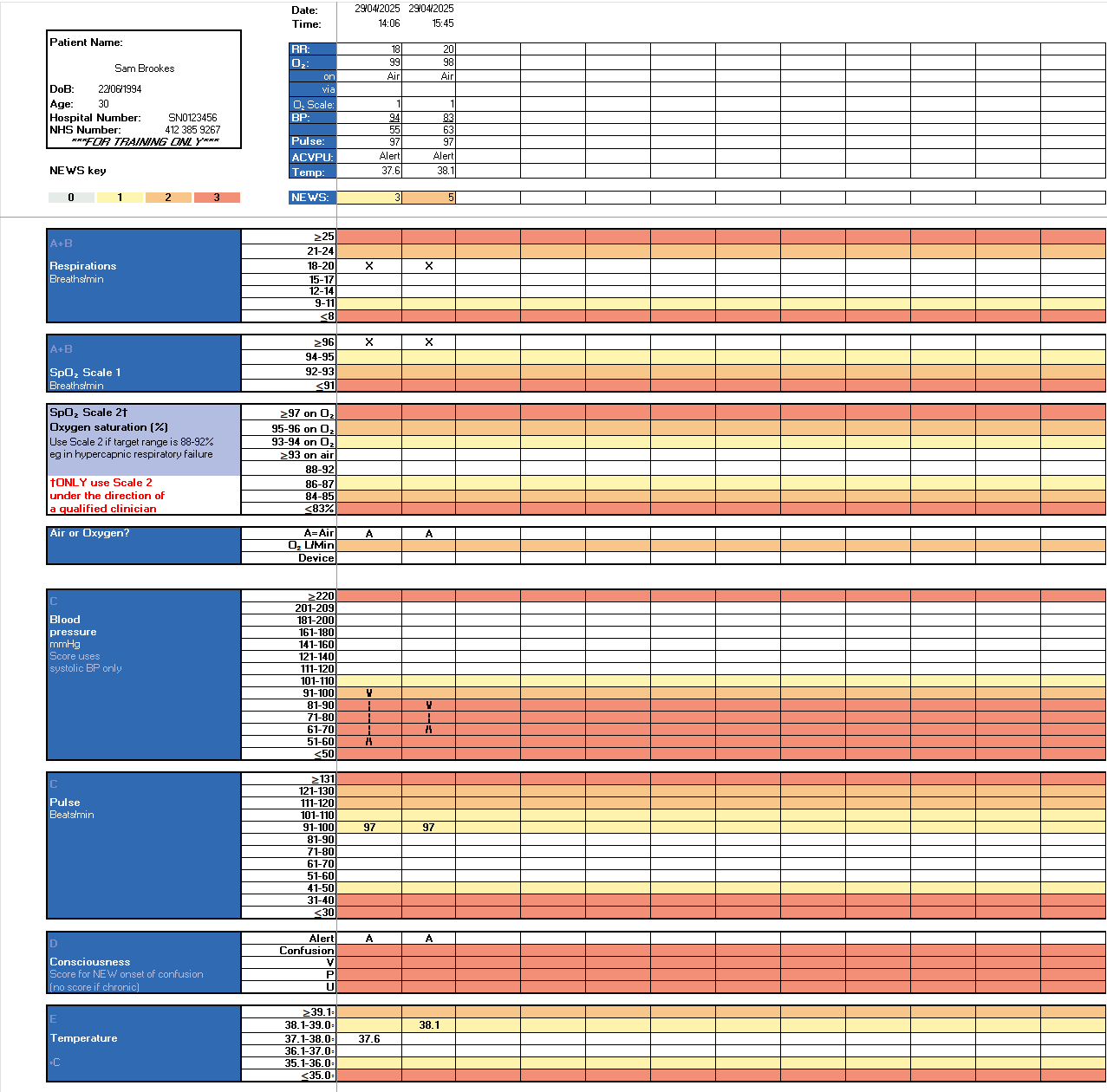
**

**C.5 Assessment Simulation – Simulated Patient Observations Post-Fluids:
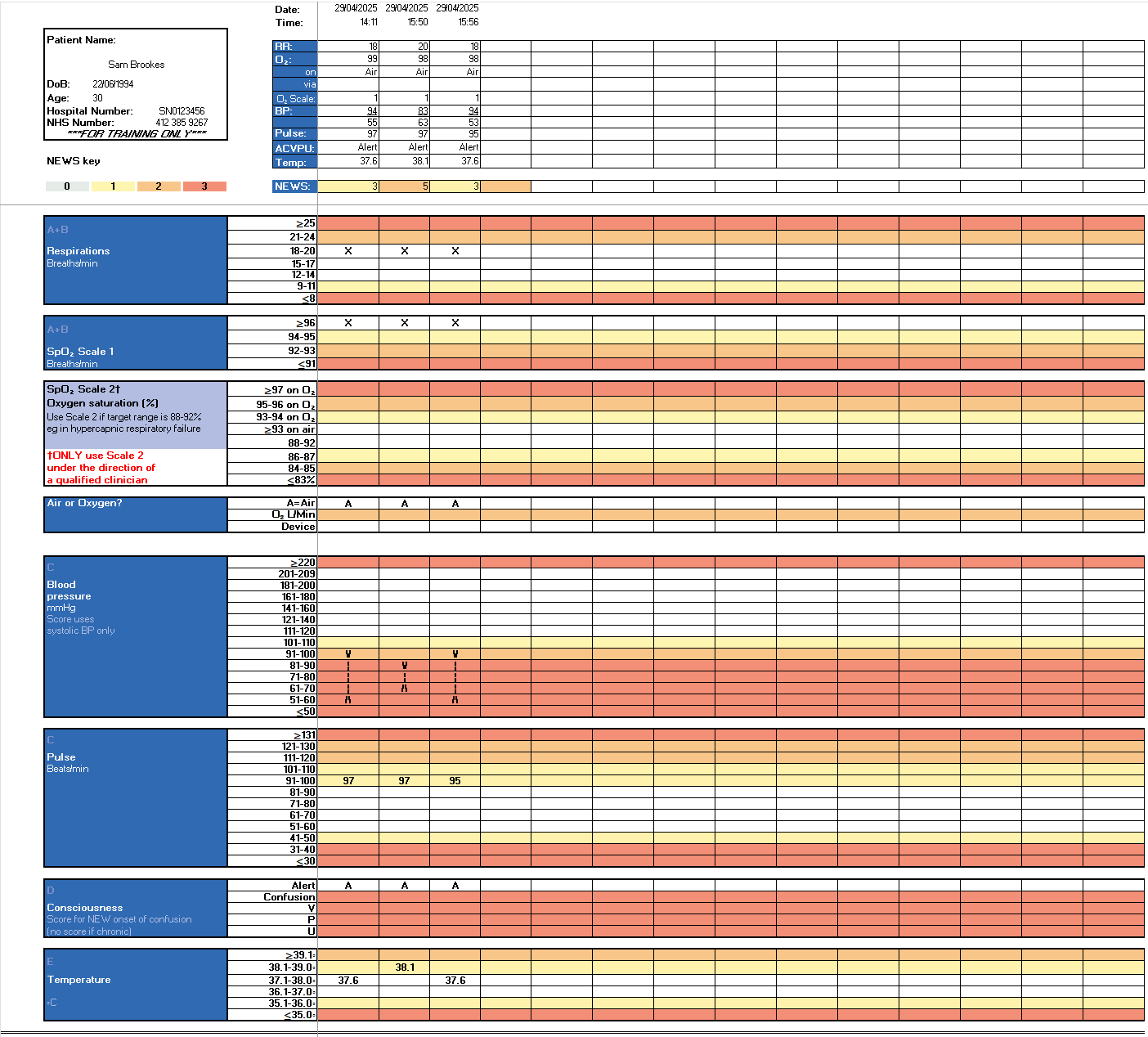
**

**
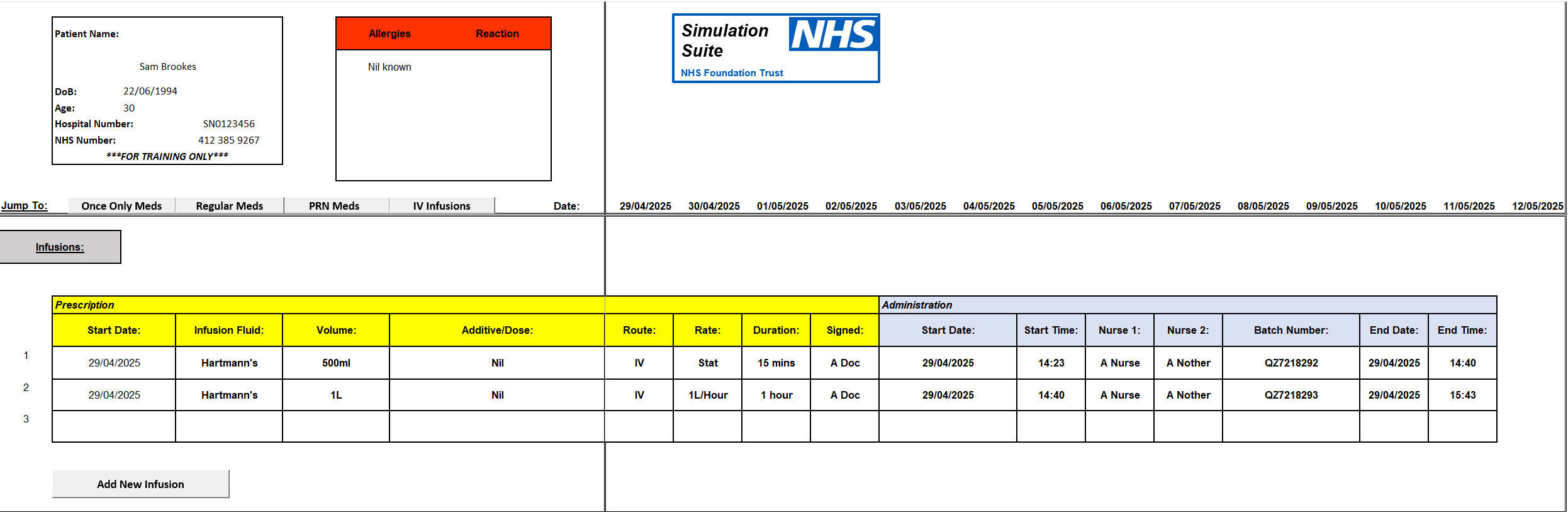
C.6 Assessment Simulation – Simulated Patient Fluid Chart (Given Prior to Scenario Start):**

**C.7 Assessment Simulation – Simulated Patient Venous Blood Gas:**

**
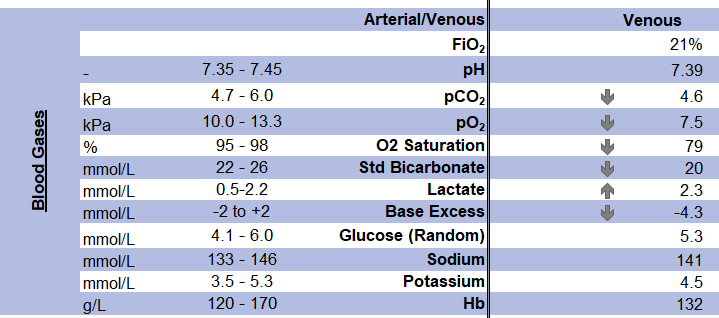
**

**C.8 Assessment Simulation – Modified Queen’s Simulation Assessment Tool (mQSAT)**

**Station - Septic Shock**

Study ID: Date of Assessment:

Assessor:­­­­­­­­­­­­

| **Primary Assessment** | | | | |
| --- | --- | --- | --- | --- |
| *Washes hands Checks patient ID*  *Checks main complaint Measures/checks vital signs* | | | | |
| *1*  *INFERIOR*  Delayed or incomplete performance of all criteria | *2*  *NOVICE*  Delayed or incomplete performance of many criteria | *3*  *COMPETENT*  Delayed or incomplete performance of some criteria | *4*  *ADVANCED*  Competent performance of most criteria | *5*  *SUPERIOR*  Efficient and rapid performance of all criteria |

| **Diagnostic Actions** | | | | |
| --- | --- | --- | --- | --- |
| *Takes relevant history Performs relevant examination (e.g. full A-E assessment)*  *Blood cultures Lactate (e.g. venous blood gas)*  *Urine output monitoring Other Bedside tests – ECG, urine dip*  *Other blood tests – FBC, CRP, U&E, LFT, Imaging – Chest x-ray*  *Coag* | | | | |
| *1*  *INFERIOR*  Delayed or incomplete performance of all criteria | *2*  *NOVICE*  Delayed or incomplete performance of many criteria | *3*  *COMPETENT*  Delayed or incomplete performance of some criteria | *4*  *ADVANCED*  Competent performance of most criteria | *5*  *SUPERIOR*  Efficient and rapid performance of all criteria |

| **Therapeutic Actions** | | | | |
| --- | --- | --- | --- | --- |
| *IV fluid resuscitation (up to 2L or 30ml/kg) Supplemental O_2_ if needed, targets appropriate saturations*  *IV antibiotics Monitors response of vital signs to treatments given*  *Recognises need for inotropes/vasopressors* | | | | |
| *1*  *INFERIOR*  Delayed or incomplete performance of all criteria | *2*  *NOVICE*  Delayed or incomplete performance of many criteria | *3*  *COMPETENT*  Delayed or incomplete performance of some criteria | *4*  *ADVANCED*  Competent performance of most criteria | *5*  *SUPERIOR*  Efficient and rapid performance of all criteria |

| **Communication** | | | | |
| --- | --- | --- | --- | --- |
| *Introduces self and explains clinical situation Responds appropriately to history (e.g. notes confusion)*  *Prioritises tasks and anticipates further steps Escalation to appropriate senior*  *Logically structured handover (e.g. SBAR)* | | | | |
| *1*  *INFERIOR*  Delayed or incomplete performance of all criteria | *2*  *NOVICE*  Delayed or incomplete performance of many criteria | *3*  *COMPETENT*  Delayed or incomplete performance of some criteria | *4*  *ADVANCED*  Competent performance of most criteria | *5*  *SUPERIOR*  Efficient and rapid performance of all criteria |

| **OVERALL PERFORMANCE** | | | | |
| --- | --- | --- | --- | --- |
|  | | | | |
| *1*  *INFERIOR*  All skills require significant improvement | *2*  *NOVICE*  Most skills require moderate or significant improvement | *3*  *COMPETENT*  Some skills require moderate improvement | *4*  *ADVANCED*  Some skills require minor improvement | *5*  *SUPERIOR*  Few, if any skills require only minor improvement |

**C.9 Assessment Simulation – Post-Scenario Questionnaire**

What is your overall diagnosis for this scenario? (Please be as specific as possible)

Which investigation and management steps were most relevant to this scenario?

|  | **Investigation** | ***Tick*** | **Management** | ***Tick*** |
| --- | --- | --- | --- | --- |
| **Airway** | - |  | Adrenaline  Nasopharyngeal airway  Oropharyngeal airway  2222 call/Intubation |  |
|  |  |  |  |  |
|  |  |  |  |  |
|  |  |  |  |  |
| **Breathing** | Arterial Blood Gas  Chest X-ray  CTPA  Peak Expiratory Flow Rate |  | Adrenaline  Furosemide  Nebuliser (e.g. salbutamol/ipratropium)  Oxygen  2222 call/Ventilation |  |
|  |  |  |  |  |
|  |  |  |  |  |
|  |  |  |  |  |
|  |  |  |  |  |
| **Circulation** | Baseline Bloods (FBC, U&E, LFT, CRP)  Blood cultures  Catheter/fluid balance  ECG  Troponin  Transfusion Samples  Venous Blood Gas/Lactate |  | Adrenaline  Blood transfusion  Cannula  IV Fluids  2222 call/Vasopressors/Inotropes |  |
|  |  |  |  |  |
|  |  |  |  |  |
|  |  |  |  |  |
|  |  |  |  |  |
|  |  |  |  |  |
|  |  |  |  |  |
| **Disability** | Capillary Blood Glucose  CT Head  Neurological Observations |  | Dextrose  Naloxone  2222 call/Intubation |  |
|  |  |  |  |  |
|  |  |  |  |  |
| **Exposure/**  **Other** | Abdominal X-Ray  Plain X-ray (limb, joint etc.)  Pregnancy Test  Urine Dip/Culture |  | Antibiotics  Blanket/Warming Device |  |
|  |  |  |  |  |
|  |  |  |  |  |
|  |  |  |  |  |
| **Not Listed Above (please write):** |  | |  | |

Where would be the most appropriate place to care for this patient (pick 1)?

Standard ward/Level 0………………………………………………………………………………..☐

Standard ward with enhanced observation/Level 1………………………………………… ☐

*e.g. at risk of deterioration but manageable on ward with support from Critical Care Outreach team*

High Dependency Unit/Level 2……………………………………………………………………..☐

*e.g. single organ support, monitoring/nursing needs above level 1*

Intensive Care Unit/Level 3…………………………………………………………………………. ☐

*e.g. advanced respiratory support (intubation/ventilation), multiple organ support*

If above Level 0, please state the reason/indication:
