# Supplementary material for: Virtual Reality Versus In-Person Simulation of Sepsis for Medical Students: Randomized Comparative Pilot Study
Source: JMIR Med Educ. 2026 Mar 30;12:e80316. doi: 10.2196/80316 (PMC13035032; doi:10.2196/80316)
Supplement: Multimedia Appendix 2 [file mededu-v12-e80316-s002.docx]

Multimedia Appendix 2 - Baseline Testing

Table S1: Number of Hours Prior In Person Simulation Experience

| **Study Group** | **0-4h** | **4-8h** | **8-12h** | **12-16h** | **16-20h** | **20-24h** | **24-28h** | **28-32h** | **32-36h** | **36-40h** | **40+h** |
| --- | --- | --- | --- | --- | --- | --- | --- | --- | --- | --- | --- |
| IP-VR-Assess | 0 | 0 | 4 | 0 | 2 | 1 | 0 | 0 | 0 | 0 | 1 |
| VR-IP-Assess | 0 | 0 | 0 | 1 | 0 | 0 | 0 | 1 | 0 | 0 | 0 |
| IP-Assess | 0 | 1 | 2 | 2 | 1 | 1 | 1 | 0 | 0 | 0 | 0 |
| VR-Assess | 0 | 1 | 0 | 2 | 0 | 0 | 2 | 0 | 0 | 0 | 0 |
| Values indicate number of students in each category | | | | | | | | | | |  |

Table S2: Number of Hours Prior Virtual Reality Medical Simulation Experience

| **Study Group** | **0-4h** | **4-8h** | **8-12h** | **12-16h** | **16-20h** | **20-24h** | **24-28h** | **28-32h** | **32-36h** | **36-40h** | **40+h** |
| --- | --- | --- | --- | --- | --- | --- | --- | --- | --- | --- | --- |
| IP-VR-Assess | 7 | 0 | 0 | 0 | 0 | 0 | 0 | 0 | 0 | 0 | 1 |
| VR-IP-Assess | 2 | 0 | 0 | 0 | 0 | 0 | 0 | 0 | 0 | 0 | 0 |
| IP-Assess | 6 | 1 | 0 | 0 | 1 | 0 | 0 | 0 | 0 | 0 | 0 |
| VR-Assess | 5 | 0 | 0 | 0 | 0 | 0 | 0 | 0 | 0 | 0 | 0 |
| Values indicate number of students in each category | | | | | | | | | |  |  |

Table S3: Number of Hours Prior In Person Simulation Experience

| **Study Group** | **0-4h** | **4-8h** | **8-12h** | **12-16h** | **16-20h** | **20-24h** | **24-28h** | **28-32h** | **32-36h** | **36-40h** | **40+h** |
| --- | --- | --- | --- | --- | --- | --- | --- | --- | --- | --- | --- |
| IP-VR-Assess | 2 | 5 | 1 | 0 | 0 | 0 | 0 | 0 | 0 | 0 | 0 |
| VR-IP-Assess | 1 | 1 | 0 | 0 | 0 | 0 | 0 | 0 | 0 | 0 | 0 |
| IP-Assess | 1 | 7 | 0 | 0 | 0 | 0 | 0 | 0 | 0 | 0 | 0 |
| VR-Assess | 4 | 1 | 0 | 0 | 0 | 0 | 0 | 0 | 0 | 0 | 0 |
| Values indicate number of students in each category | | | | | | | |  |  |  |  |
