# Supplementary material for: Virtual Reality Versus In-Person Simulation of Sepsis for Medical Students: Randomized Comparative Pilot Study
Source: JMIR Med Educ. 2026 Mar 30;12:e80316. doi: 10.2196/80316 (PMC13035032; doi:10.2196/80316)
Supplement: Multimedia Appendix 3 [file mededu-v12-e80316-s003.docx]

Multimedia Appendix 3 - Sensitivity Analyses

Table S1: Summary of mQSAT Scores by Study Group - Mean (SD)

| **mQSAT Domain** | **IP-Assess** | **IP-VR-Assess** | **VR-Assess** | **VR-IP-Assess** | **p-Value** |
| --- | --- | --- | --- | --- | --- |
| Primary | 4.15 (0.78) | 3.86 (0.96) | 4 (0.71) | 3.8 (0.85) | 0.921 |
| Diagnostic | 3.5 (0.28) | 3.73 (0.64) | 3.5 (0.24) | 3 (0.28) | 0.165 |
| Therapeutic | 3.55 (0.64) | 3.77 (0.58) | 4.08 (0.59) | 3.2 (0.28) | 0.876 |
| Communication | 3.5 (0.71) | 3.82 (0.77) | 3.75 (0.35) | 3 (0.85) | 0.788 |
| Global | 3.4 (0.42) | 3.73 (0.51) | 3.83 (0.47) | 3 (0.28) | 0.552 |
| Total | 18.1 (2.55) | 18.91 (3.34) | 19.17 (2.12) | 16 (2.26) | 0.495 |
| SD: Standard Deviation. p-Value by Analysis of Co-Variance (First mQSAT Score as Covariate) | | | | | |

Table S2: Summary of mQSAT Scores by Study Group - Mean (SD)

| **mQSAT Domain** | **IP-Assess** | **IP-VR-Assess** | **VR-Assess** | **VR-IP-Assess** | **p-Value** |
| --- | --- | --- | --- | --- | --- |
| Primary | 3.15 (0.07) | 3.18 (0.13) | 3.17 (0.24) | 3.3 (0.14) | 0.652 |
| Diagnostic | 3.3 (0.28) | 3.27 (0.64) | 3.33 (0.47) | 3.1 (0.14) | 0.143 |
| Therapeutic | 3.3 (0.14) | 3.32 (0.19) | 3.33 (0) | 3.3 (0.14) | 0.986 |
| Communication | 3.35 (0.35) | 3.41 (0.19) | 3.25 (0.12) | 3.5 (0.42) | 0.369 |
| Global | 3.35 (0.21) | 3.41 (NA) | 3.42 (0.12) | 3.4 (0.28) | 0.627 |
| Total | 16.45 (0.92) | 16.41 (1.61) | 16.5 (0.71) | 16.6 (0.85) | 0.428 |
| SD: Standard Deviation. p-Value by Analysis of Co-Variance. Analysis using unblinded reviewer scores only | | | | | |

Table S3: Summary of mQSAT Scores by Study Group - Mean (SD)

| **mQSAT Domain** | **IP-Assess** | **IP-VR-Assess** | **VR-Assess** | **VR-IP-Assess** | **p-Value** |
| --- | --- | --- | --- | --- | --- |
| Primary | 3.65 (0.86) | 3.52 (0.93) | 3.58 (0.8) | 3.55 (0.79) | 0.652 |
| Diagnostic | 3.4 (0.52) | 3.5 (0.75) | 3.42 (0.5) | 3.05 (0.56) | 0.143 |
| Therapeutic | 3.42 (0.56) | 3.55 (0.51) | 3.71 (0.59) | 3.25 (0.38) | 0.986 |
| Communication | 3.42 (0.69) | 3.61 (0.71) | 3.5 (0.43) | 3.25 (0.78) | 0.369 |
| Global | 3.38 (0.48) | 3.57 (0.52) | 3.62 (0.39) | 3.2 (0.59) | 0.627 |
| Total | 17.27 (2.17) | 17.66 (3.09) | 17.83 (2.25) | 16.3 (2.44) | 0.428 |
| SD: Standard Deviation. p-Value by Analysis of Co-Variance. Analysis using all four reviewer scores | | | | | |

Table S4: Recognition of Septic Shock and Need for Critical Care Referral by Study Group

| **Number of Prior Debriefs** | **Study Group** | **Simulation Modality** | **N** | **Septic Shock Recognised** | **Septic Shock p-Value** | **Critical Care Recognised** | **Critical Care p-Value** |
| --- | --- | --- | --- | --- | --- | --- | --- |
| 1 | IP-Assess | VR | 10 | 7 | 0.838 | 1 | 0.000 |
| 1 | VR-Assess | IP | 6 | 5 |  | 3 |  |
| 2 | IP-VR-Assess | VR | 11 | 7 | 0.914 | 2 | 0.247 |
| 2 | VR-IP-Assess | IP | 5 | 3 |  | 0 |  |
| p-Value by Binomial Test. Agreed by any single reviewer | | | | | | | |

Table S5: (i): Comparison of All Four Simulation mQSAT Scores for Group: IP-Assess

| **Domain** | **Reviewer 1 (LM)** | **Reviewer 2 (TO)** | **Reviewer 3 (Blinded 1)** | **Reviewer 4 (Blinded 2)** | **Krippendorff’s alpha** |
| --- | --- | --- | --- | --- | --- |
| Primary | 3.2 (0.42) | 3.1 (0.32) | 4 (0.94) | 4.3 (0.95) | -0.095 |
| Diagnostic | 3.4 (0.84) | 3.2 (0.63) | 3.3 (0.82) | 3.7 (0.48) | 0.370 |
| Therapeutic | 3.4 (0.52) | 3.2 (0.42) | 3.2 (0.63) | 3.9 (0.57) | 0.080 |
| Communication | 3.3 (0.67) | 3.4 (0.52) | 3.1 (0.88) | 3.9 (0.88) | 0.111 |
| Global | 3.5 (0.53) | 3.2 (0.42) | 3.2 (0.63) | 3.6 (0.52) | 0.114 |
| Total | 16.8 (2.1) | 16.1 (1.66) | 16.8 (3.01) | 19.4 (2.17) | 0.124 |
| Confidence Intervals by Bootstrapping | | | | | |

| Range of Agreement | Interpretation |
| --- | --- |
| $\alpha$ $\leq$ 0.2 | Slight Agreement |
| 0.2 < $\alpha$ $\leq$ 0.4 | Fair Agreement |
| 0.4 < $\alpha$ $\leq$ 0.6 | Moderate Agreement |
| 0.6 < $\alpha$ $\leq$ 0.8 | Substantial Agreement |
| $\alpha$ > 0.8 | Near-Perfect Agreement |

Table S5: (ii): Comparison of All Four Simulation mQSAT Scores for Group: IP-VR-Assess

| **Domain** | **Reviewer 1 (LM)** | **Reviewer 2 (TO)** | **Reviewer 3 (Blinded 1)** | **Reviewer 4 (Blinded 2)** | **Krippendorff’s alpha** |
| --- | --- | --- | --- | --- | --- |
| Primary | 3.18 (0.4) | 3.18 (0.4) | 3.45 (1.29) | 4.27 (1.01) | -0.128 |
| Diagnostic | 3.55 (1.04) | 3 (0.63) | 3.64 (0.5) | 3.82 (0.98) | 0.159 |
| Therapeutic | 3.36 (0.67) | 3.27 (0.79) | 3.82 (0.6) | 3.73 (1.01) | 0.443 |
| Communication | 3.45 (0.52) | 3.36 (0.5) | 3.64 (1.21) | 4 (1) | 0.146 |
| Global | 3.6 (NA) | 3.18 (0.6) | 3.82 (0.75) | 3.64 (0.81) | 0.323 |
| Total | 16.82 (2.48) | 16 (2.41) | 18.36 (3.88) | 19.45 (4.18) | 0.140 |
| Confidence Intervals by Bootstrapping | | | | | |

| Range of Agreement | Interpretation |
| --- | --- |
| $\alpha$ $\leq$ 0.2 | Slight Agreement |
| 0.2 < $\alpha$ $\leq$ 0.4 | Fair Agreement |
| 0.4 < $\alpha$ $\leq$ 0.6 | Moderate Agreement |
| 0.6 < $\alpha$ $\leq$ 0.8 | Substantial Agreement |
| $\alpha$ > 0.8 | Near-Perfect Agreement |

Table S5: (iii): Comparison of All Four Simulation mQSAT Scores for Group: VR-Assess

| **Domain** | **Reviewer 1 (LM)** | **Reviewer 2 (TO)** | **Reviewer 3 (Blinded 1)** | **Reviewer 4 (Blinded 2)** | **Krippendorff’s alpha** |
| --- | --- | --- | --- | --- | --- |
| Primary | 3.17 (0.41) | 3.17 (0.41) | 3.83 (0.41) | 4.17 (1.17) | -0.155 |
| Diagnostic | 3.67 (0.52) | 3 (0) | 3.33 (0.52) | 3.67 (0.82) | 0.103 |
| Therapeutic | 3.33 (0.82) | 3.33 (0.82) | 3.83 (0.75) | 4.33 (0.82) | 0.491 |
| Communication | 3.17 (0.41) | 3.33 (0.52) | 3.5 (0.55) | 4 (0.89) | 0.297 |
| Global | 3.5 (0.55) | 3.33 (0.52) | 3.67 (0.52) | 4 (0.89) | 0.436 |
| Total | 16.83 (1.6) | 16.17 (1.94) | 18.17 (2.04) | 20.17 (3.82) | 0.267 |
| Confidence Intervals by Bootstrapping | | | | | |

| Range of Agreement | Interpretation |
| --- | --- |
| $\alpha$ $\leq$ 0.2 | Slight Agreement |
| 0.2 < $\alpha$ $\leq$ 0.4 | Fair Agreement |
| 0.4 < $\alpha$ $\leq$ 0.6 | Moderate Agreement |
| 0.6 < $\alpha$ $\leq$ 0.8 | Substantial Agreement |
| $\alpha$ > 0.8 | Near-Perfect Agreement |

Table S5: (iv): Comparison of All Four Simulation mQSAT Scores for Group: VR-IP-Assess

| **Domain** | **Reviewer 1 (LM)** | **Reviewer 2 (TO)** | **Reviewer 3 (Blinded 1)** | **Reviewer 4 (Blinded 2)** | **Krippendorff’s alpha** |
| --- | --- | --- | --- | --- | --- |
| Primary | 3.4 (0.55) | 3.2 (0.45) | 4.2 (0.84) | 3.4 (1.14) | -0.003 |
| Diagnostic | 3.2 (0.84) | 3 (0.71) | 3.2 (0.84) | 2.8 (0.84) | 0.451 |
| Therapeutic | 3.4 (0.55) | 3.2 (0.45) | 3.2 (0.45) | 3.2 (1.1) | 0.387 |
| Communication | 3.6 (0.55) | 3.4 (0.55) | 2.8 (0.84) | 3.2 (1.48) | 0.055 |
| Global | 3.6 (0.55) | 3.2 (0.45) | 3 (0.71) | 3 (1.22) | 0.152 |
| Total | 17.2 (1.64) | 16 (1.87) | 16.4 (3.21) | 15.6 (5.37) | 0.229 |
| Confidence Intervals by Bootstrapping | | | | | |

| Range of Agreement | Interpretation |
| --- | --- |
| $\alpha$ $\leq$ 0.2 | Slight Agreement |
| 0.2 < $\alpha$ $\leq$ 0.4 | Fair Agreement |
| 0.4 < $\alpha$ $\leq$ 0.6 | Moderate Agreement |
| 0.6 < $\alpha$ $\leq$ 0.8 | Substantial Agreement |
| $\alpha$ > 0.8 | Near-Perfect Agreement |
